# Improving Resilience in Times of Multiple Crisis: Commentary from a German Economic Policy Point of View

**DOI:** 10.1007/s41471-022-00150-y

**Published:** 2022-12-20

**Authors:** Andreas Pinkwart, Gideon Schingen, Anna-Tina Pannes, Dirk Schlotböller

**Affiliations:** 1 Innovation Management and Entrepreneurship, HHL Leipzig Graduate School of Management, Leipzig, Germany; 2 State Parliament of North Rhine-Westphalia, Platz des Landtags 1, 40221 Düsseldorf, Germany; 3 Düsseldorf, Germany; 4 Düsseldorf, Germany; 5 Düsseldorf, Germany

## Abstract

Due to the trend towards more frequent and multiple crises, it is essential for enterprises as well as for states and entire economic areas to develop strategies to improve resilience. In this article, existing challenges and conceivable strategies for improving resilience will be outlined from a microeconomic as well as from a macroeconomic perspective. To mitigate vulnerabilities of Supply Chains in the last decades, drivers for improving Supply Chain resilience have been explored. They are presented as contributing to building resilience on the micro level. On the macro level, opportunities and limits of current approaches for strategic independence are discussed. In terms of a new regulatory policy, a rescue of honour for global cooperation and free trade is proposed by the authors.

## Introduction: Post-euphoric Era of Worldwide Trade and the Importance of Resilience

The “End of History” has not yet come. The ideal of a worldwide dominance of liberal democracies based on a free market economy as stipulated by the US-American political scientist Francis Fukuyama in his essay (Fukuyama 1989) did not come true. This prediction was once fertilized by the overarching optimism in times of epochal changes when the existing bipolarity of the cold war came to an end. However, it has been refuted by the reality of new conflict lines and system competition. Not only has the COVID-19 pandemic challenged the global trading system, it has also interrupted supply chains and has mercilessly revealed existing dependencies. Even before the outbreak of the Russian war of aggression against Ukraine, raising security issues with a kind of painful brutality, the pandemic already had a popular feature in political and economic debates to become the motif of longings: resilience.

Resilience means the adaptation and bouncing back after a disruptive event, a stressful event or a shock. In material sciences a substance is said to be resilient, when it turns back to its original form after its deformation (Brunnermeier 2021). Psychology defines resilience as the individual resistance, the strength of which is essential to the duration and success of the recovery after a stress-inducing or an arduous event (Flüter-Hoffmann et al. 2018). At the level of collective actors, resilience describes the ability of an organisation to react to a disturbance jeopardizing the status quo. In this regard, after a disruption, a resilient enterprise returns to business as usual or alternatively, to a status which is expected to be expedient (Flüter-Hoffmann et al. 2018; Fiksel et al. 2015). Furthermore, entire economies can be affected by pivotal disruptive events and can prove to be more or less resilient when coping with the consequences. In economics, shocks can be described as abrupt and unforeseen changes in the growth dynamics of one scale (Brunnermeier 2021). Shocks differ in the probability of their occurrence, in their amplitude which means their deviation from the normal status and in their frequency. Moreover, they can occur over different time frames: transitory shock (e.g. a short-term inflation surge) on the one hand, only has a temporary effect. On the other hand, a persistent shock (e.g. climate change) changes the basic conditions in the long-term.

Contrary to what the one-sidedly connoted language use suggests, shocks can be differentiated with regard to the evaluation of their positive or negative impact. In fact, regarding the tremendous consequences of the COVID-19 pandemic to health and economy, using the well-known term of a “crisis as a chance” does not appear to be appropriate. However, it is also part of this analysis to describe the positive effects of the findings. In this regard, the effect of a “digital booster” can be described as a development driven by the pandemic on which it could be further built upon in economics, education and administration (Pinkwart and Pannes 2022). This provides an example for a positive component of progress: Resilience is not only defined as the ability to bounce back and to return to the original “status-quo-ex-ante”. In the contrary, it can also be understood as the capability to assimilate change and to take advantage of the absorbed change by turning challenges into opportunities and creating superior performance than before (Jüttner and Maklan 2011; Hohenstein et al. 2015; Heredia et al. 2022).

Moreover, resilience opens a path to the future as no crisis is singular in its effects nor will be the last one. For actors in economics and politics, it is of utmost importance to be as well prepared as possible in future. Resilient economies and enterprises are asked to develop strategies and mechanisms how to cope with shocks and crisis. In this way, they can create a basis for an optimistic view of the future. However, the debate on resilience does not only focus on whether enterprises or entire economies can bounce back and can once recover but also on the question whether learning experiences can induce a new oscillation, bounce back and recover once but also on the question whether learning experiences can cause the new oscillation to proceed in an even more agile and better prepared manner (Fiksel et al. 2015). The process of responding to shocks will depend on the extent and novelty of the event as well as on the degree of preparation and adaptation (Sheffi and Rice 2005). If the degree of preparation is high, the process of responding to shocks can be characterised as *pro-active*, if the degree is low, its response will lead to a lack of *improvisation*. On the other axis of a 2 × 2 matrix we can measure the degree of adaptation. If this degree is low, the process of responding will be rigid, if it is high, the process works in an agile way. Four different configurations can be set up along these two axes (Burnard et al. 2018). The first quadrant is defined by non-active preparation and rigid adaptation. Consequently, the process of responding works “*at high risk*”. On the diagonally opposite side (fourth quadrant), the “*resilience focused*” configuration is characterised by a good balance between robust planning for expected shocks and allocating the necessary resources needed to overcome the unexpected. While the “*process based*” configuration in the second quadrant describes systems responding in a way that is based on a robust planning for expected shocks and combined with a low degree of adaptation towards overcoming the unexpected, the so called “*resourceful*” configuration characterises systems with a high need to improvise in combination with many available resources to overcome the crisis (Burnard et al. 2018).

In this context, resilience begins with preparing for a future crisis. According to (Martin 2012), who analysed the reaction of a recessive shock to regional resilience, four different dimensions of resilience can be defined covering the different time frames from the operative to the strategic crisis management. Accordingly, the first dimension of resilience covers the *resistance* in form of load-bearing capacity and adaptability of the existing system in response to a shock. The second dimension focuses on *recovery* as capability to retrieve the efficiency of a system to a certain level for a certain period of time. A third dimension of resilience is *re-orientation* in the sense of a new development and orientation of a system as a means to avoid negative consequences by future shocks. The last dimension of resilience is *renewal* which means to make fundamental changes to obtain a form of sustainable resilience.

At present, the resistance of sole enterprises and entire economies and economical regions is of utmost importance and resilience is challenged in many respects:^1^

- In a short period of time, the COVID-19 pandemic caused a shock to people and the economy that is still reverberating. It refers to risks through external shocks which can be hardly influenced as for example, natural disasters which will occur more likely in the future with the progression of climate change.
- Internationalization is currently relapsing. Instead, protectionism takes over, the competition of systems with China evolves and the Russian war of aggression against the Ukraine painfully shows our economical vulnerability and dependency.
- Climate change and the management of its consequences demands resilience in a broad sense. It is about creating chances to preserve habitats worth living.
- The digital transformation is accompanied by several factors that challenge resilience. Fundamental technological skills and infrastructures need to be protected against dependencies and cyberattacks. Business models according to “the-winner-takes-it-all”-principle (Frank and Cook 1996) that are built on digital platforms as well as a development towards monopolization of platforms and infrastructure create dependencies and make technology leadership more relevant.

Facing the increasing trade conflicts, the recently experienced challenges that pandemics and natural disasters have imposed on societies and companies, the century-long task to achieve a climate-neutral lifestyle as well as ethical questions when it comes to cooperation with autocracies, a post-euphoric era of world trade can be postulated. This raises important open questions to Germany and Europe that have yet to be answered.

Due to the trend towards more frequent and multiple crises (Albach et al. 2015), it is essential for enterprises as well as for states and entire economic areas to develop strategies to improve resilience. In the following, existing challenges and conceivable strategies for improving resilience will be outlined from a microeconomic as well as from a macroeconomic perspective. To mitigate vulnerabilities of supply chains in the last decades, drivers for improving supply chain resilience have been explored. They are presented as contributing to building resilience on the micro level. On the macro level, opportunities and limits of current approaches for strategic independence are discussed. In terms of a new regulatory policy, a rescue of honour for global cooperation and free trade will be proposed in the last section.

## Symptoms of Our Times: Crises, Conflicts and Climate Change

### The Pandemic as a Sign of Our Vulnerability

The colloquial and economic use of terms with regard to the COVID-19 pandemic match very closely: The rapid spread of the COVID-19 virus as well as the subsequent consequences caused a “shock”. The pandemic represents an exogenous and unexpected natural disaster influencing almost every economic area and affecting the entire global economy. It is also a kind of a stress test of resilience in its essence. The pandemic uncovered an enormous vulnerability in many different areas and relentlessly revealed lacks in preparation and risk protection. Important supply chains broke, and bottlenecks are still noticeable. The Corona virus paralyzed the entire world and showed the fragility of the system of global labour division. Interestingly, some areas have, however, proven to be remarkably flexible and were quickly re-established after a short period of adjustment time.

Most likely, an overall assessment would show that German industry and labour market have proven to be a reasonably resilient economy in times of the pandemic. Manufacturing output slumped 31.5% from February 2020 (a six-month high) to April 2020 alone—and had already recovered 87%, or 27.3 percentage points, of that by the end of the year. Employment subject to social security contributions fell by 1.2% from February to May 2020, but had already reached a new high peak by May 2022 (Destatis 2022). Before the Russian war of aggression against Ukraine, it seemed foreseeable that Germany could return to pre-pandemic levels and to approach the previous growth curve. Most other economies have reached pre-crisis levels more quickly, but usually at the cost of higher infection and death rates. How resilient this is compared to other strategies needs to be weighed. In price-adjusted terms, global trade volumes already exceeded pre-crisis levels again in October 2020, and global container throughput even reached pre-crisis levels as early as the summer—at least in the interim (Baur and Flach 2022). Government support is likely to have prevented major distortions. Moreover, financial aid for the economy and short-time working allowances significantly increased government debt. However, the crisis also revealed various weaknesses and problems.

The impact of the various closures and disrupted supply chains caused a shock to businesses that was felt keenly even in situations where there was no large financial loss. Without preparation, several activities had to be transferred to a digital format, enterprises had to develop protective measures and to reorganize working processes within short time. Suddenly, several activities had to be transferred to a digital format, enterprises had to develop protective measures and to reorganize working processes within short time. The vulnerability experienced as an actor in a globalized and extremely labour divided economy must be used as a trigger for future long-term changes beyond the pandemic (Pinkwart and Pannes 2022). In the area of politics and administration, the pandemic has made clear the countries’ need for modernization and revealed a remarkable lack of resilience. Manually maintained health data sent by fax has become a symbol of our outdated administration. The German state and especially the German health system were poorly prepared for a pandemic (Gleißner and Follert 2022). This was also the case for the entire EU. It was caused by a lack of adequate crisis awareness and by a distribution of responsibilities which was unclear or unnecessarily complicated.

Considering that resilience, as initially described, is not only about coping with a virulent event but also about strengthening the future “bounce back capabilities”, learning from experiences that can be used as material for risk prevention become of fundamental importance. Even if the Corona pandemic reaches an endemic stage, another virus will probably become relevant by zoonotic transmission, or a more dangerous variant can occur from a known virus. Moreover, other disasters that are beyond human influence, such as natural disasters or terrorist attacks on infrastructure of systemic importance have to be included in risk analysis and in robust planning.

### The Current Energy Crisis as a Revelation of Insufficient Resilience

For years, the German energy policy has been committed to switching from fossil and conventional energy sources to wind, solar and other renewables for power generation. To compensate for the loss of secured power during the transition, gas was chosen as a temporary solution. In addition, the use of gas promised less greenhouse gas emissions and was classified by the EU-Commission as sustainable energy form. At the same time, the number of gas supplying sources decreased due to the drying out of German and Dutch gas sources and the conscious decision not to open up new gas or shale gas sources in own or neighbouring regions and due to the decision not to build own LNG terminals. However, the issue of LNG-terminals has always been a key issue in German energy policy for a long time. The German Monopoly Commission (Monopolkommission 2002) referred to LNG-terminals as a back-stop technology to secure supply security at a time when the German government decided to outsource supply security to a highly concentrated private gas industry (Hellwig, 2022a and 2022b; Gehrig and Stenbacka 2022). With the liberalization of the gas market and the frequent realignment of the German energy policy the gas demand steadily increased and with it also the energy dependency of Russia grew. Moreover, the interconnection at the latest was extended to property rights of pipelines and storage infrastructure. Facing the significant political tensions after the annexation of Crimea in 2014 and the imposed sanctions, a supply of Russian gas could not have been regarded as really secured. In response to the Luekex 18 exercise, the responsible office published a report in 2019 (Federal Office for Civil Protection and Disaster 2019). The Federal Council also called on the German government to increase the resilience of the energy system, especially in the gas sector, by taking specific precautionary measures (Federal Council 2019). Even though the government experienced a crisis situation, no adequate measures were taken to enable a flexible and robust response in case of a real crisis (less preparation). The same applies to the preparation of the technical requirements for a possible announcement of an emergency level by the network agency.

As a result, the German energy system, and thus the entire national economy, was massively disrupted by the outbreak of the Russian war against Ukraine in 2022. By this, the process of responding started time-delayed in the light of the war from a resilience configuration “at high risk”. This is characterised by improvisation and in some case also rigid adaptation. Actions in such a situation are highly prone to explosively increasing prices and shortcomings as they are discussed for example by Hellwig (2022a). The short-term replacement of Russian gas for ongoing processes and the storage through liquid gas imports confront gas wholesalers as well as industries with extreme price jumps. It is of utmost importance for the federal government to procure sufficient substitutes for Russian gas and to diversify the sources in the short and medium term to restore the security of energy supply and to recover economy. Therefore, the federal government supports the energy sector when it comes to contract negotiations with important liquid gas suppliers by organising global energy purchasing tours. In parallel, new regulations for fast development of the LNG terminals and their connection to the national gas network were introduced at accelerated proceeding. This is in contrast to other measures, like the reactivation of conventional power plants (nuclear and coal) that already have been shut down or are scheduled to be closed or the governmental allowance to produce the industrial heat temporarily by a fuel switch gas to oil and coal. However, it is assumed, that these measures will contribute to secure the energy supply and to mitigate the price explosion (Pinkwart 2022). Due to this rigidity, the reallocation of resources towards overcoming the shock started with delay (Grimm 2022).

The high share of Russian gas in the German energy demand also aggravated the cluster risk for energy wholesalers such as Uniper. Uniper purchases more than half of its gas stocks from Russia on the basis of long-term contracts (systemic risk). The urgent demand makes it necessary to stabilize the energy supply and to limit the energy price explosion, whatever is necessary. This is accompanied by a huge financial burden: 8 months after the outbreak of the war, the government launched a stabilization fund with a volume of up to 200 billion euros. An independent ‘gas-commission’ of experts draw up proposals on how households and companies could be most effectively relieved from this fund of the dramatically increased energy costs. The primary goals are to expand the gas supply, to reduce its demand and to broaden sources of gas supply.

In reaction to the acute lack of gas and the enormous increase in energy costs, there exist various market and political incentives to strongly expand renewable energies on-shore and off-shore and to globally promote the production of hydrogen (re-orientation). It shows some similarities with the concept of *ambidexterity*, which is recommended to stabilize a fundamental transformation process by balancing the exploitation of current conventional resources and technologies and the exploration and implementation of radical new ones (Tushman and O’Reilly 1996). An accelerated expansion of the transmission grids at macro and micro levels is planned to provide renewable energy from all parts of Europe in a more efficient and secured way and should now be implemented without delay. At the same time, a stronger decentralization of the energy supply in the respective residential and commercial areas together with digital tools, such as smart metering and energy sharing, will help to transform energy on side and to store it in a more flexible manner. Thereby, possible energy forms are energies obtained by heat pumps, photovoltaic, wind or hydrogen. Natural stabilizers evolve which can be further strengthened by hydropower and biomass. For the nationwide allocation of electricity and heat there exist large grids making available renewable energies as well as supplementary conventional energies throughout the EU. It is of central significance for the robustness of the entire energy system that this transformation proceeds more ‘resilience focused’ at rapid speed (Hanselka 2022; Bichler et al. 2022).

### The Networked World and Its Polarisation

Free trade is subject to many (new) restrictions in today’s networked world. Even before the pandemic, nationalism and protectionism were on the rise again in many regions of the world. The interruption of supply chains resulting from the Corona pandemic caused further disruption of trade flows. In addition, trade is also under moral pressure. The western world is questioning trade with authoritarian, non-democratic regimes. In the struggle for political and economic strength, further protectionist approaches are tested. Additionally, changes in economic size are becoming apparent: EU’s weight in the global economy is diminishing.^2^ A swan song to globalization is often sung. However, due to a lack of alternatives the end of globalization is neither proven nor promising. The discussion about “*deglobalization*” shows, however, that old certainties are critically scrutinized. If something new emerges from this, it will be fruitful. The term “deglobalization” is used to describe trends away from global free trade (Stiglitz 2021). In order to present the facets and background of the current phase of deglobalization, Stiglitz gives his impressions of this year’s World Economic Forum. They range from regulatory market restrictions (e.g. USA especially in the Trump era) to plans of increasing autonomy by strengthening the “internal” economic cycle (China). Supply chain diversification—in terms of the basal security logic of redundancy (Brunnermeier 2021)—is an essential resilience strategy that can reduce the risks of disaster-related, political and economic shocks to industries and companies.

Two perspectives can be distinguished: The first is the argumentation of free trade as such, including the ethical questions of system competition. The second is the question of one-sided dependencies that arise within free trade. The positive mechanisms of free trade in a globalized world are still valid. David Ricardo already highlighted these positive mechanisms in the 19th century, (Ricardo 1817). Trade is beneficial to the economy as a whole because the advantages of specialization and division of labour can be better utilized as “gains from trade”. In general, both parties benefit from the advantages of the division of labour. It becomes evident by the fact that transactions between individuals or nations are undertaken on a voluntary basis.^3^ Free trade makes it possible and even necessary for an economy to specialise in the production of certain goods. By exploiting economies of scale, specialization can offer additional advantages. See (Porter 1990) for the self-reinforcing role of economies of scale in industry clusters. Welfare gains can be achieved through specialization in a world based on the division of labour—each economy focuses on what it does best.

However, if an imported good is difficult to substitute (in the short term) and there is little competition, there is a *risk of dependency*. Producers may be dependent on a particular raw material (e.g. gas), a particular intermediate product (e.g. computer chips) or a single company or market (e.g. China). A dependency exists if there is no possibility of substituting partners and products when needed—this and not the number of suppliers is decisive for a dependency. In a polypolistic market (e.g. baked goods), the choice of a single supplier can also be rational because substitution possibilities exist. Especially in trade relations with foreign countries, we have recently become painfully aware of how quickly wars, pandemics, political decisions and strategic and security considerations can act as “game changers”. However, dependence on individual domestic suppliers or customers can also be fatal.

Russia’s brutal war of aggression against Ukraine raises ethical issues and questions about resilience.^4^ Germany’s dependency on Russia became dramatically visible, even though German-Russian economic relations have already suffered noticeably as a result of the Crimea conflict in 2014 and the subsequent sanctions.^5^ Above all, Russia played an extraordinarily important role as a major supplier of energy and raw material for Germany and the EU. In 2021, still 27% of German primary energy consumption was covered with gas. Of those 27% (Federal Environment Agency 2022), 55% was Russian gas (bdew 2022).

Since February 2022, politics and society have been confronted with the question of how Germany admitted self-critically that the “energy partnership” was more of an “energy dependency” (FAZ 2018), the negative effects of which Germany had been clearly warned of for some time. The fact that Russia had always kept its delivery obligations created a misleading feeling of deceptive security (FAZ 2014). The growing dependency turned out to be a mistake which was recognized far too late. This is weakening German and European resilience. Are similar mistakes being made in trade relations with China? As the questions of peace and respect for human rights of the trading partners gain importance from a resilience perspective, a respective dependency analysis is required from this.

China’s economic rise shows the efficiency of market economy mechanisms. Within a few years, China advanced to become a global player. However, the economic promises of the huge market cannot hide the fact that neither market economy nor democracy are part of the Chinese state system. The case of Hong Kong, the behaviour towards Taiwan, the brutal action against the ethnic group of the Uyghurs or the hard enforcement of the No-Covid-Strategy in Shanghai speak a clear language. The Chinese leadership clearly proclaims its will to secure a position of supremacy in the world. The “market” in which we trade with China is a politically driven economy with strong market elements.

Dependencies as a threat to resilience exist in particular with China. There are enormous risks in system rivalry, because the framework conditions can quickly change to the disadvantage of foreign investors and trading partners when it seems to be politically opportune. The dependency in sensitive areas of high technology and infrastructure is also worrying. If there are critical dependencies in the EU, they often exist with China (European Commission 2021). It is true that many of the raw material or products with a currently high import share are also available within the EU or could be manufactured here. So far, extraction has not been economically viable. However, this calculation is likely to change in future. In addition, exploiting domestic potential helps us to do without raw material that are extracted under unacceptable environmental and social conditions.

To “save the honour” of trade, it must be said, that international division of labour remains indispensable as a source of prosperity. Furthermore, trade increases the likelihood of a state’s peacefulness (Weede 2021): War between two countries becomes less likely the more they trade with each other, the higher the mutual foreign investments are and the better it is generally determined in terms of economic freedom. Cooperation in the sense of an exchange of goods thus increases the probability of peace. In this sense, the old formula “change through trade” has proven itself without being able to derive an automatism from it.

### Climate Change—Global Task of the Century

The scientific community and the majority of countries recognize climate change as the greatest challenge of our time. Climate change poses special challenges for international cooperation in which different rationalities must be balanced. There is not enough space here for an overall assessment of the logics of action and potential breaking points of cooperation. Essential for climate protection is the character of a global public good. This gives rise to two dilemmas concerning: the temporal dimension on the one hand and the interdependent dimension on the other. Climate protection measures that are taken now usually yield their “dividend” in the future on a rather abstract level (generational dilemma). The main problem, however, is that the success of measures for individuals on the path to limiting global warming cannot be felt or measured. Moreover, without further collective efforts they would be ineffective (*free rider problem*). Only the sum of all measures results in effectiveness, and in the best case all major actors act in the same way.

Natural sciences and economics clearly show that global climate neutrality is a common interest of the world community. However, because climate protection measures (currently) cause costs that tend to lead to a competitive disadvantage to a company, a pioneering role for individual states is not rational per se—this is especially true for poor and economically less developed states. Therefore, climate protection measures also influence trade. There is a threat of an imbalance between those states that want to implement the goal of climate neutrality in a binding manner by the middle of this century and take corresponding measures, and those that are not (yet) prepared to do so. Instruments such as the “Carbon Border Adjustment Mechanism” or “Carbon Contracts for Difference” discussed in the European Union are intended to ensure fair competition. However, they involve considerable measurement and monitoring efforts, are open to attack under trade law and mostly ignore exports.

A “*climate club*” as a coalition of the willing parties leading the way with binding targets, certificate trading and technology transfer, can build confidence in functioning climate protection as a growth factor. Of elementary importance for global climate protection efforts is the attractiveness of success: states working with binding targets and effective measures can give proof that the transformation is feasible and financially viable and could offer the underlying technologies on the world market. This is the only way other states can be won over to climate protection via the technological and economic incentives. Regardless of how well we succeed in protecting the climate we will face the consequences of climate change. Without question, it poses a fundamental threat to global prosperity because it threatens entire habitats. It also has an impact on trade, which is expected to intensify in future. As with pandemics, resilience for companies means anticipating and weighing the risks of climate change. These include increasing heat waves, heavy rain events, crop failures and other disasters.

## Strategies for Improving Resilience: Micro and Macro Level

### Strategies for Building Supply Chain Resilience (Micro Perspective)

With the beginning of the 80’s a new approach named “Supply Chain” found its way into the economy. Areas, such as procurement, production and distribution that in companies previously had been managed separately were interlinked and networked across the board from the customer’s point of view. Supply chain was defined as: *“the network of organizations that are involved, through upstream and downstream linkages, in the different processes and activities that produce value in the form of products and services in the hands of the ultimate consumer”* (Martin 1992). Since the 1990s the discipline of Supply Chain Management (SCM) was developed very dynamically in research and practice. Due to rapid developments in information technology and to the new wave of “business process reengineering” (Davenport and Short 1990; Fiedler et al. 1995), open markets and a massive drop in freight rates product flows from the production of raw material all the way through to the final consumer and could be optimized in a global context. For achieving more efficiency and higher speed, the integration of main elements of logistics, operations management and marketing into cross-functional inter-organizational processes was extended (Christopher and Peck 2004). Beside (further) improvements in efficiency it was possible to adapt new developments in the marketplace and to change customer requirements (Christopher and Peck 2004). SCM opened up the opportunity of leveraging synergies from the integration and management of internal and external company processes (Lambert and Cooper 2000).

#### Improving Supply Chain Resiliency as a Growing Task

Since the early beginning of the new millennium with the terroristic attack on the World Trade Center in New York (Sheffi 2001) or the SARS epidemic in 2003 in Asia, companies are increasingly confronted with unpredictable serious events caused by natural disasters, terrorism, political turmoils, wars, diseases, financial and economic crises (Sheffi and Rice 2005) as well as with technology and business model disruptions (Christensen 2013). Initial studies were the basis for the developing of new SCM fields—for instance for Supply Chain Risk Management (SCRM), and for Supply Chain Resilience (Hohenstein et al. 2015). Researchers identified an often noticed substantial disconnect in companies between the definition of the corporate strategy and the recognition of the effects of those strategic decisions upon supply chain vulnerability (Christopher and Peck 2004). By referring to the strategy of global sourcing for achieving lower unit costs it was noted that the definition of cost was often used in a very limited frame. Risks to the supply chain through extended or lead times, the dependency on business partners who themselves were possibly vulnerable to disasters or the potential loss of control were often not taken seriously into account (Christopher and Peck 2004). It was also found that managers often did not completely understand the extent of the networks of which their companies were a part of and their interrelated roles and perspectives (Lambert and Cooper 2000). Due to the fact that a regional disaster can induce a disruption with global effects on Supply Chain ecosystems, collaborative and coordinated approaches in public-private partnerships at an international level were recommended (World Economic Forum 2008). Given the macro-economic and microeconomic impact of supply disruptions arising from a range of global risks, improved dialogue and policy on these risks is crucial to the effective management of the global economy. Although there is no control over the cause, a company has the responsibility to mitigate them (Calvo et al. 2020). A substantial analysis of the Supply Chain networks with regard to their systemic risks and vulnerabilities (Christopher and Peck 2004) was also missed.

These developments called for more resiliency in Supply Chain (Hohenstein et al. 2015). Consequently, this fields of SCRM and SCRES received an increasing attention in recent years. Whereas SCRM aims at identifying and reducing the vulnerability of risks for the Supply Chain, SCRES focuses on the developing of adaptive capabilities to be prepared for unexpected events and to respond to disruptions and recover from them (Jüttner et al. 2003; Ponomarov and Holcomb 2009; Jüttner and Maklan 2011). The growing importance of this topics finds its visible expression in a large number of publications and an equally increasing number of literature reviews (Hohenstein et al. 2015; Calvo et al. 2020; Han et al. 2020). Starting from a common definition of resilience “*as the ability of a system to turn to its original state or move to a new, more desirable state after being disturbed” *(Christopher and Peck 2004), Supply Chain Resilience can be defined as the Supply Chain’s ability to prepare for unexpected events (readiness phase), respond to disruptions (response phase) and to turn to its original operations at the desired level of connectedness and control over structure and function (recovery phase) or to move to a new, more desirable state after being disturbed (growth phase) (Ponomarov and Holcomb 2009; Jüttner and Maklan 2011). In order to develop and evaluate strategies for more resiliency in Supply Chain it is necessary (i) to analyse the relevant vulnerabilities of SC, (ii) to identify the right drivers for resiliency in SC, (iii) to learn more about the right balance of different actions in different situations and environments for improving resiliency and (iv) to establish appropriate metrics for measuring their effectiveness before, during and after the disturbance (Sheffi and Rice 2005; Hohenstein et al. 2015).

#### Potential Supply Chain Vulnerabilities

The vulnerability of a Supply Chain to a specific risk varies considerably depending on the company concerned and the environment in which it operates. It is highest when both the likelihood and the impact of disruption are high. Those characterized by low probability but high impact call for robust planning and a response that goes well beyond normal day-to-day actions (Sheffi and Rice 2005). The following Supply Chain vulnerability factors have been identified and categorized as follows (Pettit et al. 2010; Sheffi and Rice 2005; Fiksel et al. 2015; Hohenstein et al. 2015): There are vulnerabilities caused by changes in the business environment that are beyond a company’s control (*turbulences*), as there are geopolitical disruptions, natural disasters and pandemics but also shifts in customer demand. Another kind of vulnerability are *intentional threats, *such as terrorism, sabotage, cyber-attacks or strikes. Additional vulnerabilities came from *external pressure *that not specifically targeting the company but create constraints or barriers, such as competitive innovations, government regulations and price pressures as well as shifts in cultural, social and environmental attitudes. In addition, there are *limited resources, *such as the availability of gas in the current energy crisis or of special materials like semiconductor ships (Pettit et al. 2010). Relevant fields of vulnerabilities are also the importance of high *sensitivity*, such as carefully controlled conditions for product and process integrity, the complexity of the production process and the degree of *connectivity*, such as the dependence and reliance on sources and information flow or the degree of outsourcing (Pettit et al. 2010)*. *Furthermore, Supply Chains are vulnerable to risks that may occur with their multiple tiers of customers and suppliers in the Supply Chain. Finally, the examples of the aforementioned vulnerabilities show that they can reinforce each other. This is especially true if the necessary countermeasures are taken too late or not decisively enough.

#### Capabilities to Improve the Resilience of Supply Chains

Consistent with (Wieland and Wallenburg 2013), resilience can be both proactive and reactive in nature. Therefore, in literature main SCRES drivers are grouped in proactive and reactive capabilities. But there are also studies (Hohenstein et al. 2015) showing that import capabilities like flexibility and redundancy work both proactively and reactively and in all four phases.

*Flexibility* is an enabler for better preparedness as well as to adapt and to adjust to a disruption rapidly and to move faster to a new and more desirable state after the disruption (Teece et al. 1997; Jüttner and Maklan 2011; Wieland and Wallenburg 2013; Fiksel et al. 2015). Over the past decades, companies and business economics have achieved manifold progress in better understanding and increasing flexibility. Furthermore, the informational revolution creates new opportunities for companies to adjust to changes with greater flexibility (Albach et al. 2015). Initiatives to improve flexibility could be the development of multi-skilled workforce, a production system that can accommodate multiple products, and real-time changes and a sourcing strategy that permit the transparent switching of suppliers (Rice and Caniato 2003). Whereas *redundancy* includes safety stock, maintaining excess capacity, dual sourcing (Gehrig and Stenbacka 2022) backup sites and the strategic use of excess resources (Sheffi and Rice 2005; Wieland and Wallenburg 2013).

*Visibility* refers to the use of information technology to have access to or to share timely information about Supply Chain operations and to promote transparency of information and awareness of the current situation in the Supply Chain (Fiksel et al. 2015; Jüttner and Maklan 2011). *Situation awareness* is the capability to recognize and predict a potential disruption; such a capability requires knowledge of Supply Chain vulnerabilities and information sharing (Han et al. 2020). *Collaboration* concerns the exchange of information and the application of shared knowledge to decrease uncertainty and increase visibility and customer service (Sheffi and Rice 2005). Data-sharing arrangements can help to enable both pre-emptive resilience building and effective crisis management (World Economic Forum 2008). *Agility* is the capability to respond quickly to unpredictable changes in market demand or supply by adapting Supply Chain processes (reconfiguration, reduce lead time and reduce non-value action), as customer requirements are constantly changing. (Christopher and Peck 2004; Han et al. 2020).

*Velocity* refers to the speed with which a Supply Chain can react to unexpected events and market changes (Christopher and Peck 2004; Jüttner and Makan 2005). *Knowledge management* is the capability to learn from feedback of a disruption to develop better plans and solutions for future ones (Ponomarov and Holcomb 2009). *Contingency planning* improves recoverability by evaluating processes such as Supply Chain reconfiguration, scenario analysis, and resource reconfiguration. (Birkie et al. 2017; Ponomarov and Holcomb 2009). Last not least,* market position* refers to the financial outlook, including financial strength, market share and loss absorption (Fiksel et al. 2015). For example, a strong market position with a high market share opens up the possibility of making more investments in SCRE. (Sheffi and Rice 2005). Based on empirical findings, Gehrig (2015) emphasized that the strategic role of capital for a more resilient banking system is also related to the institutional environment. The positive effect of capital is stronger in countries with stronger banking competition and more pronounced insolvency risk.

#### Finding the ‘Zone of Balanced Resilience’

As shown by (Datta et al. 2007), any previously presented single resilience capability factor when applied in isolation under uncertainty can also degrade the performance. Although introducing redundancy can help mitigate the risk of unwarranted events (Sheffi 2001; Sheffi and Rice 2005), too much redundant inventory can cause problems, as excess inventory can lead to complacency (Datta et al. 2007). While investing in redundancy is purely a cost increase, investing in flexibility brings many additional benefits and operational efficiencies in the normal course of business (Sheffi and Rice 2005). Flexibility not only strengthens an organization’s resilience, but also creates a competitive advantage in the marketplace. Using the automotive industry as an example, Sheffi and Rice (2005) explain that it makes little sense for car manufacturers to set up redundant production facilities worldwide. Instead, it is more advantageous for them to keep production flexible enough to meet regional requirements so that, if necessary, vehicles can also be manufactured for other regional markets. They conclude that making Supply Chains more flexible has a much greater leverage effect than increasing redundancy.

Therefore, a balanced approach for concrete strategies is recommended by adjusting the different capability factors used by different actors of the Supply Chain for managing Supply Chain risk und resilience effectively. In their case based study Fiksel et al. (2015) identified linkages between specific vulnerabilities and capabilities which can be used to build proactive strategies for the improvement of resiliency. For using these opportunities in practice they developed an assessment tool for businesses, which they call Supply Chain resilience assessment and management (SCRAM). The tool was tested in collaboration with more than twenty business units of the global company Dow Chemical and delivered positive results. The SCRAM approach helped the managers to improve the understanding of the inherent vulnerabilities that could lead to disruptions and the capability factors that are within their control. The company could reduce the frequency of disruptions and the severity of their impacts, resulting in increased customer satisfaction and reduced Supply Chain operating costs. In the field of tension between low/high degree of vulnerabilities and low/high degree of capabilities the managers have to find the ‘zone of balanced resilience’ (Fiksel et al. 2015), where the portfolio of capabilities is matched to the concrete structure of vulnerabilities.

Contrary to the often heard opinion of managers, Birkie et al. (2017) found out, that increased Supply Chain complexity does not have a detrimental effect in an acute crisis. On the contrary, a certain level of complexity has been tested as beneficial for better recovery of operational performance affected by the disruption. Resilience capabilities become more effective when more resources and higher levels of complexity are used in the Supply Chain (Birkie et al. 2017). In contrast, Wieland and Wallenburg (2013) found out that forms of relationships that rely on tight integration of processes and systems between Supply Chain members cannot address vulnerabilities in the Supply Chain to a greater extent than in loose types of relationships. This can partly be explained by referring to the role of resource dependencies (Wieland and Wallenburg 2013). As suggested by Sydow (2015), network research in general should pay more attention to the ‘dark side of networks’ in order to better understand how organizational persistence and path dependencies arise with this supposedly flexible form of government. With regard to redundancy, the most current study by Gehrig and Stenbacka (2022) provides an elementary argument for dual sourcing as an investment into supply security. By analyzing the strategic sourcing decisions of the individual firm in a partial equilibrium setting they come to the result that dual sourcing helps to curb short-term opportunistic behavior by providers with significant market power even if the dual sourcing technology is more expensive than the preferred source. It could be shown by the authors that this argument holds even when the supply capacities of the dual source are not actively used while in operation (Gehrig and Stenbacka 2022).

### Strategic Independence (Macro Perspective)

Particularly in the state sector, a lack of resilience was lamented during the Corona pandemic. In the health sector, for example, the lack of FFP2 masks was seen as evidence of deficiencies. Providers in the health sector are usually organized under private law, although they are closely regulated by the state. In fact, security of supply is a dimension of resilience. For example, stock-keeping can ensure, at least temporarily, that the population can be adequately supplied with food, energy and medicine in a crisis. The reflex to call for stock-keeping is obvious. It is therefore not surprising that Germany has a Federal Grain Reserve (“Bundesreserve Getreide”) and a Civil Emergency Reserve (“Zivile Notfallreserve”). At considerable cost, almost one million tonnes of grain and lentils are stored for emergencies (Rudloff 2022). A smallpox reserve (“Pockenreserve”) of 100 million vaccine doses is also being stored since the early 2000s. However, the food reserve could only supply a small part of the population for a short period of time—a switch to ready-made food was rejected with reference to its high costs (German Federal Parliament 2020). Especially after the experiences in the Corona pandemic, the question remains whether a complex “vaccination through” would actually be logistically feasible in a sufficiently short time in the event of a terrorist attack on Germany with smallpox viruses. This, and also the fact that the emergency reserves have never been needed show that the government measures to ensure supply security in the event of a disaster at least have a symbolic character.

The EU provides another example of the quest for security of supply. In the past, it has expended considerable effort to become independent of food imports from abroad and to achieve strategic autonomy. In the process, the European Common Agricultural Policy, introduced in 1962, pursued a price support policy in the following years to achieve its goal (German Federal Parliament 2020). As soon as a certain market price was undercut, the EU bought agricultural products to stabilize the price. This policy was quite effective: The EU changed from a net importer to a net exporter of food in the 1970s and 1980s. The guaranteed intervention price made it attractive for producers to increase supply. As a consequence, it led to the well-known “butter mountains” and “milk lakes” for which no appropriate use could be found. The effects on the EU budget were also considerable—in the 1970s, the common agricultural policy consumed 90% of the total EEC budget.

The experience with state stock-keeping and with state intervention in the economic incentive system to secure supplies has often been mainly expensive and of modest benefit. Now, insurance is not superfluous just because one has not made use of it. Nevertheless, it remains unclear whether the insurance would really have been serviceable and helpful in case of need. The term “resilience” often remains so unspecific that it can be applied to a multitude of different risks. No two crises are the same. Therefore, it is less about concrete measures than about patterns and decision-making capacity. Even state stock-keeping could not have done anything against the 2008 financial crisis. In Germany, medical protective equipment is stored in a “National Health Reserve” since the Corona pandemic (Die Zeit 2021)—but it is uncertain whether there will be another comparable pandemic in the coming decades and whether medical protective equipment will then be in short available again.

In this sense, general measures for a *robust state* are certainly the best guarantee for more resilience (Gleißner 2020). The state does not have to be prepared for every eventuality, but must be able to make the necessary decisions quickly and flexibly in the event of a crisis (Gleißner and Follert 2022). A good regulatory framework that allows and promotes an efficient, flexible and diversified economy protects against dependency. A sustainable budgetary policy also allows for support measures when they are needed: Germany was only able to launch the generous aid programmes in the Corona pandemic because it had the corresponding budgetary leeway. Conversely, government debt that is out of balance with economic performance challenges resilience in the long run, for example through significant debt crises, massive tax increases or high inflation that is not consistently fought. At the same time, a resilient public spending policy requires a clear prioritization on investments—unlike in the past (from 2000–2019, public net investments were negative on average). Finally, the Ukraine conflict shows that the danger of war is by no means averted on the European continent. A robust state therefore also requires Germany to restore its defence capabilities enabling it to defend itself and its allies adequately in a timely manner.

If the goal of resilience is to be pursued beyond these rather general measures it must first be defined for which areas state resilience would be required at all—this is especially true since very different crises are conceivable. To do this, it is necessary to operationalise the term and the desired level accordingly—concrete goals and measures must be named and, if necessary, coordinated with each other (Rudloff 2022). For some years now, the EU has begun to develop resilience strategies in response to the 2010 financial crisis, see the European Strategy and Policy Analysis System (ESPAS 2015). For a systematic crisis assessment, see European Commission (2020). There, economic-social, ecological, geopolitical and digital resilience are named as strategic core objectives (Rudloff 2022). Stress tests can also be carried out and, if necessary, it must be clarified how to deal with the results. Within the framework of its industrial strategy, the EU Commission has, for example, identified 137 out of 5200 products with a high dependence on imports—production in the EU would be significantly more expensive or impossible to establish in the short term (European Commission 2021). The Commission has identified a need for action especially in raw materials and chemicals for energy-intensive industries, for the digital and green transformation, and for healthcare. Here, companies are in demand above all, but also politicians. In the medium and long term, the expansion of trade agreements and the dismantling of trade restrictions are helpful. In the short term, the suspension of tariffs and the dismantling of punitive tariffs on shortage products can also be helpful (Treier and Herweg 2022).

An alternative European approach can be well illustrated by the European Chips Act, which was publicly discussed in particular due to the high investments announced by Intel in Magdeburg and Bosch in Dresden, among others (European Commission 2022). The EU share of the one trillion-euro chip market was around 10% in 2020. Especially in view of the strategic importance of computer chips in the value chain and the strong but exposed position of Taiwan, which is confronted with Chinese territorial claims, this share is perceived as too low. With investments of 43 billion euros, the European world market share is to be increased to 20% in 2030.

The dismantling of trade restrictions or targeted state support for strategically important industries show that state resilience does not necessarily mean that the state itself ensures the supply of the population and the economy. It always makes sense to examine the extent to which market actors can be integrated into a resilience strategy—if necessary with corresponding legal requirements. Examples can be found in Switzerland or Great Britain, where emergency partnerships are concluded in which supermarkets commit themselves to storage and delivery services in crisis situations (Rudloff 2022). The German Control and Transparency Act (Kontroll- und Transparenzgesetz) successfully obliges limited companies (AGs and GmbHs) to implement early warning systems, which can also be understood as a state-imposed resilience measure for companies (Wellbrock 2022). Voluntary agreements between the private sector and the state that reward desired behaviour also offer a good way forward. In Germany, on the other hand, there was serious discussion during the pandemic about confiscating strategically important goods—the suppliers of these goods would thus have been punished.

Due to the interdependencies of economic cycles, which are difficult to oversee, and the unspecific risk addressed by resilience, a major challenge for the state is to identify and name possible bottlenecks and crises in time and to initiate countermeasures. Good experience has been gained with monitoring systems, for example. The “Agricultural Market Information System”, for example, was founded by the G20 to be better prepared for food supply crises such as those in 2008 and 2011. It provides transparent information on the risks to the economy or a sector.^6^ The system is also linked to the international policy level. By referring to the information gathered, fewer export restrictions were imposed on agricultural products during the Corona pandemic than had been feared (Rudloff 2022). Such a politically desired and transparent monitoring system could also be applied to critical goods and infrastructures. In 2021, the G7 actually proposed similar monitoring for minerals and metals, which should also be interwoven with the political level. This refers to the Critical Minerals and Metals Information System (CriMMIS) (Rudloff 2022).

Moreover, the establishment of monitoring systems at the intergovernmental level shows that resilience goes beyond the supply goals of individual states. A modern concept of resilience therefore not only encompasses the security of supply of one’s own population, but also takes into account the effects and interdependencies on the international trade (Rudloff 2022). Autonomy in the sense of national security of supply, on the other hand, should not be the goal of a resilience strategy.

Resilience also cannot mean that the state tries to take all the risks away from private actors. It is more important to create the right framework conditions and to rely on individual and market coordination and self-healing forces—a lean state is better able to act in serious crisis situations. Above all, it is essential to avoid serious mistakes—such as misguided incentives for private individuals: For example, prices should be understood as scarcity signals that must also have an effect. Bureaucratic, undifferentiated attempts at detailed control can significantly limit flexibility and responsiveness. The socialisation of risks can be tempting in the short term but highly problematic in the long term, for example if companies can pass on the negative consequences of misjudgements to the state. Insurance always carries a moral hazard risk. An example would be an excessively long and state financed short-time work without employers’ co-payment (Felbermayr 2022): In the interim, short-time work is a proven instrument for ensuring resilience to economic cycles, but in the long term there is a risk of burdening the insured community with wrong decisions in corporate policy, such as too little inventory.

## Prospects: Ideas for an International Regulatory Framework

The proportion of people living in extreme poverty has been falling for decades (World Bank 2022). International division of labour and trade are likely to have contributed significantly to this and are probably a good strategy for global prosperity in the long term. Therefore, it seems premature to write off the international division of labour and globalization and the thesis of “*change through trade*”. However, the current trade distortions—with discussions on de-globalization and near-shoring and, on the other hand, China’s two-cycle economy—clearly point to existing conflicts in the current global order. The opinion that one’s own country is not benefiting sufficiently from global trade seems to be accepted in many cases.

One way to address international trade conflicts is to partially renegotiate the current rules for world trade and to bring about reforms. In international institutions, such as the WTO, there is certainly a need for reform: At present, restrictions on the export of goods are possible almost unconditionally according to WTO standards, which regularly leads to problems, especially in food crises. See (Karapinar 2010) for a critical appraisal. And even in resource-poor Germany, export bans on timber were discussed when shortages became apparent during the Corona pandemic. The EU’s “export authorisations” for medical products were actually implemented at the beginning of the pandemic.

Beyond the undoubted need for reform, international organizations have another inherent problem: a good set of international rules alone does not automatically lead to fair world trade. To be effective, trade rules must be followed and enforced where necessary. The “enforcement” of a rule is understood in the following to mean that it persists unaffected by formal changes and is applied in such a way that its intended goals are achieved (Vanberg 2011). In the international context, this means that trade agreements must be self-enforcing: adherence to rules must be permanently in the mutual interest of all participants, because there is no “world government” that could monitor trade agreements and enforce them (Weingast (2005); Weingast (1997); Barzel (2000); Ordeshook (1992) and Niskanen (1990)). Only in this way will international agreements be respected even without enforcement power (Niskanen 1990). The core proposition of modern constitutionalism in political economy is that a social order must be in everyone’s interest and every individual must have an interest in its observance (Przeworski 2006). Self-enforcing rules can only be realised in the international context through strong international institutions. As in a club, the members of an international trade organization have far-reaching rights such as exemption from customs duties, the dismantling of non-tariff trade barriers, common standards or the mutual recognition of patent rights. In return, the “club members” submit to the jurisdiction of the respective institution in international trade matters. The latter mediates compromises where possible—and, where necessary, international trade law is interpreted via strong arbitration tribunals. The credible threat of exclusion from international benefits and conventions as a last resort is at best sufficient to ensure enforcement of the common rules.

If new answers to global questions can be found with the help of international institutions, this would also be a contribution to greater resilience: When it comes to limiting CO2 emissions, for example, national efforts have no chance of success due to their limited contribution. Vague self-restrictions and appeal are unlikely to lead to success—at least globally. If, on the other hand, common reduction targets can be agreed upon internationally, an international organization can be the guarantor of efficient target achievement: In a “climate club”, to which the most important global players would have to belong, a uniform system for CO2 pricing could be agreed upon—ideally it would be a global trade in certificates. However, the agreed rules would also have to be self-enforcing for the international “climate club”. Participation in CO2 pricing must therefore be permanently in the mutual interest of all participants. It would therefore be clever to link certificate trading with the privileges of a strong international trade organization: As long as membership of an international trade organisation outweighs its disadvantages—e.g. through certificate trading—compliance with international agreements is in the mutual interest. Members that violate agreed environmental requirements run the risk of also being excluded from the trade benefits of the organization. In their totality, international trade and climate treaties would be mutually beneficial and thus self-enforcing.

Climate policy is a good example of global coordination problems, but by no means the only one. Child labour, human rights, environmental pollution and resource depletion are other fields that the global community must address together. If strong international institutions succeed in establishing global and self-enforcing rules, more and not less international cooperation would be the solution for more resilience and fair world trade. The fact that in 2021 only 46% of the world’s population would still be living in some form of democracy also shows impressively that cooperation on global issues must also take place with states that do not meet western standards of democracy and morality (Economist Intelligence Unit 2021). If solutions to global problems are to be found, international cooperation with as many states of the world as possible is urgently needed.

## Footnotes

1 Resilience is also challenged on an individual level. For example, if crises require more flexibility on the job or individual crises may challenge the psychological resilience. However, the individual level is not in the focus of this paper.

2 The share of today’s EU-27 countries in global gross domestic product adjusted for differences in purchasing power has fallen from 20 to 15% between 2001 and 2021, the share of the USA from 20 to 16%. At the same time, China’s share has risen from 8 to 19% (IMF database, as of 16.09.2022).

3 Within an economy, foreign trade can nevertheless also create winners and losers, as already shown by the Stolper-Samuelson theorem, cf. (Stolper and Samuelson 1941). However, appropriate lump-sum transfers allow for Pareto improvements.

4 Accordingly, “sanctions” in the sense of a trade boycott also always affect both sides. If one side can exercise market power, it will skim off a higher rent—but also has more to lose in the case of an embargo.

5 Exports to Russia have fallen by a good quarter since the annexation of Crimea by 2021, while imports have decreased by 20%. The effect of sanctions is empirically disputed: presumably, the expectation of future trade is more important than good past relations. In this respect, sanctions undermine the peace induced by capitalism. Neither Russia nor China would certainly accept human rights just because sanctions were imposed; cf. Weede (2021) or also Felbermayr (2022). In terms of an opportunity cost consideration, however, lost profits can certainly be relevant to decision-making and increase future credibility.

6 The Agricultural Market Information System was founded by the G20, for example, to be better prepared for agricultural price crises such as those in 2008 and 2011. The system is interlinked with the international political level (Rapid Response Forum).
